# Health-Seeking Behaviors and Self-Care Practices of People with Filarial Lymphoedema in Nepal: A Qualitative Study

**DOI:** 10.1155/2015/260359

**Published:** 2015-01-28

**Authors:** Ram Kumar Adhikari, Jeevan Bahadur Sherchand, Shiva Raj Mishra, Kamal Ranabhat, Amrit Pokharel, Pramila Devkota, Durga Mishra, Yadu Chandra Ghimire, Khageshwor Gelal, Rajan Paudel, Rajendra Raj Wagle

**Affiliations:** ^1^Department of Community Medicine and Public Health, Institute of Medicine, Tribhuvan University, Kathmandu, Nepal; ^2^Manmohan Memorial Institute of Health Sciences, Lalitpur, Nepal; ^3^Nepal Development Society, Chitwan, Nepal; ^4^National Public Health Laboratory, Department of Health Services, Ministry of Health and Population (MoHP), Kathmandu, Nepal; ^5^Kathmandu Medical College Teaching Hospital, Kathmandu, Nepal; ^6^UNDP, Lalitpur, Nepal; ^7^Department of Health Services, Ministry of Health and Population, Kathmandu, Nepal

## Abstract

*Background*. Lymphatic filariasis is endemic in Nepal. This study aimed to investigate health-seeking behaviors and self-care practices of people with filarial Lymphoedema in Nepal. *Methods*. A cross-sectional study was conducted using qualitative methods in three endemic districts. Twenty-three patients with current Lymphoedema were recruited in the study. *Results*. Hydrocele was found to be a well-known condition and a major health problem in the studied communities. People with Lymphoedema primarily sought health care from traditional healers, whereas sometimes home-based care was their first treatment. Later Ayurvedic and allopathic hospital-based care were sought. Respondents reported various psychological problems such as difficulty in engaging in sexual intercourse, anxiety, worry and stress, depression, low self-esteem, feeling weak, fear of being abandoned, and fear of transmitting disease to the children. Standard foot care practices except washing were largely absent. * Conclusions*. Lymphoedema in the limbs and hydrocele were found to be major health problems. The traditional health care providers were the first contact of care for the majority of respondents. Only a few patients had been practicing standard foot care practices.

## 1. Background

The World Health Organization (WHO) considers lymphatic filariasis (LF) the second leading cause of physical disability worldwide [1, 2]. Of the 1.4 billion people who lives in filaria endemic areas in the 73 endemic countries, 120 million people are currently infected [2]. The most common chronic manifestations of the disease are Lymphoedema and hydrocele [3]. Globally, there are 15 million people with Lymphoedema and 25 million men with urogenital swelling, principally scrotal hydrocele [2].

The chronic manifestation of Lymphoedema has a significant impact on quality of life [4]. The chronic attacks in the form of acute filarial adenolymphangitis caused by the death of adult filarial worms and acute dermatolymphangioadenitis (ADLA) due to secondary infection are common in Lymphoedema. Repeated acute episodes spur the progression from Lymphoedema to Elephantiasis and have greater short term disability [5]. Morbidity management of Lymphoedema slows the progression, relieving pain and discomfort. A regimen of rigorous foot care practices with skin hygiene and simple self-help measures, such as limb elevation, exercise, use of topical antibiotics, and antifungals, aimed at minimizing episodes of acute dermatolymphangioadenitis (ADLA) attacks and lymph stasis is the model recommended by the World Health Organization for the management of filarial Lymphoedema [6, 7].

Filarial Lymphoedema has various psychological impacts on individual and family life. Approximately six percent of married persons experienced sexual and marital problems [8]. People with higher grades of Lymphoedema and hydrocele had more severe psychosocial problems than physical ones [9]. They are at higher risk of depression [10]. A study from Sri Lanka reported individuals suffering from chronic Lymphoedema were depressed (8.5%), felt shy (33.3%), had fear of Lymphoedema (7.3%), and perceived a major problem affecting their lives (61.8%) [8]. Suicidal intention has also been reported among people with filarial Lymphoedema [9].

LF is a public health problem in Nepal. The disease is prevalent in the rural areas of the country predominantly affecting the poorer sector of the community [11]. The gravity of the problem lies in 60 out of 75 districts being endemic and more than 25 million individuals are at risk. Prevalence rates above 20% were found in 11 districts (with the highest rate of 40%), those of 6–19% were found in 15 districts, and 0.1–5% were in 7 districts [12]. The Government of Nepal is committed to eliminating LF by 2020 [11] initiating the mass drug administration (MDA) in the Parsa district in 2003, which was later expanded to 46 districts in 2011 [11]. This programme is focused on interrupting parasite transmission by employing annual, community-wide mass drug administration [13, 14]. However morbidity management of Lymphoedema is largely neglected.

Information and insights on health seeking behaviors and self-care practices of Lymphoedema in Nepal remain meager. The objective of this qualitative paper is to explore specific health beliefs, health-seeking behaviors, and self-care practices of people with Lymphoedema in Nepal for designing socially acceptable and culturally compatible prevention and morbidity management strategies.

## 2. Methods

### 2.1. Study Setting, Tools, and Data Collection

An exploratory study was carried out in the three of sixty LF endemic districts in Nepal from July to September, 2013 [11]. The selected districts were Dhading (Salyantar Village Development Committee (VDC)), Kapilvastu (Maharajgunj VDC), and Kailali (Pahalbanpur and Malakheti VDCs). They represent the LF endemic districts in the Terai and Hill regions of Nepal. VDCs are the smallest politico-administrative units.

This study is a part of the larger study conducted on “Parasitological and sociocultural aspects of lymphatic filariasis in Nepal.” Patients with filarial Lymphoedema were purposively selected. The cases of Lymphoedema were mapped with the help of female community health volunteers and health workers in the study sites. The detailed sampling procedure is explained elsewhere [4].

A semistructured interview schedule was prepared after considering previous studies as works of references [15–17] and authors' experiences in the field. The developed tool was then reviewed by a panel of experts from the Department of Community Medicine and Public Health at the Institute of Medicine, Kathmandu, Nepal. Questions were translated into Nepali, pretested on a sample of Lymphoedema patients in the Salyantar VDC of the Dhading district, and later modified to correspond to the cultural setting of the study site.

Qualitative study techniques were used [18, 19]. In-depth interviews (IDIs) were conducted by research assistants who spoke the local language fluently. At the end of the interview, all patients were also educated on Lymphoedema management practices recommended by the World Health Organization. A total of 23 IDIs were held in three districts. Each interview lasted 45–60 minutes and was tape recorded and later translated into English. Over 200 pages of typed verbatim transcripts were obtained from audio tapes of in-depth interviews.

### 2.2. Data Analysis

**Figure 1:**
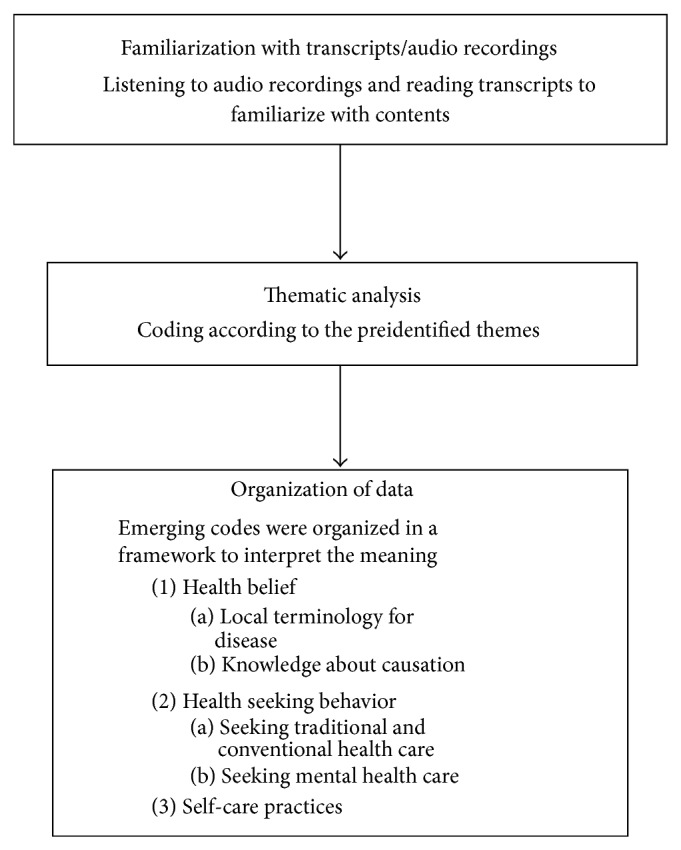
Data analysis process.

This study involved a deductive approach for content analysis [20]. A similar analysis framework has been used in Nepal before [21]. The transcript was saved in Microsoft Word 2007. Data were systematically examined for emerging codes and patterns and were divided into a priori themes: health beliefs, health seeking behaviors, and self-care practices for data analysis. This was followed by indexing, charting, mapping, and interpretation (Figure 1).

### 2.3. Research Ethics

This study obtained ethical clearance from the Institutional Review Board at the Institute of Medicine, Tribhuvan University, Nepal. Written informed consent was obtained from each respondent before taking the interview.

## 3. Results

### 3.1. Health Beliefs

#### 3.1.1. Local Terminology for the Disease

The respondents used different terminologies to refer to Lymphoedema. The most popular among them was “*Hattipaile*,” literally meaning Elephantiasis. This word is used to signify someone who has swollen legs that resemble the legs of an elephant. While “*Hattipaile*” was the most commonly used word in Dhading and Kailali districts, “*Godfuluwa*” was used in Awadhi-speaking communities in Kapilvastu. We did not find any local terminology used to signify Lymphoedema of hands in Kapilvastu and Kailai districts. However, in the Dhading district, people used “*Hatfuluwa*” and compound terminologies like “*Hatma Hattipaile bhako”.*

#### 3.1.2. Knowledge about the Causation

Knobs, lesions, and skin folds in the body, right leg swelling, left leg swelling, swelling of both the legs, and swelling of single or both arms were the major signs found. Hydrocele was found to be a well-known condition and a major health problem in the study communities. Patients and other key informants were asked what they believed to be the cause of Lymphoedema. Respondents reported that the cause of disease was related to their past work and physical activity, insect/mosquito bites, and bodily abnormality. A woman with recent ADLA attacks said,

*“… I got the disease at the age of 18 after marriage. I don't know the actual cause. Although people say that the mosquito bite causes it, I believe it is due to the defect of my internal body. I also suffer from fever, swelling and tingling of nerves and once I got unconscious for 2-3 days due to a high grade fever.”*

Others associated diseases with itching, wounds and infection, massage/contact, inheritance, excessive sexual activity, “dirty blood,” trauma, trapped gas, and witchcraft were reported by respondents to be the causes of Lymphoedema. A few of the respondents reported that prevention could be achieved by taking Diethyl Carbamazine (DEC), using a mosquito-nets and chemical spray. A man with Lymphoedema for more than 10 years said,

*“… I used to massage other people and must have contracted the disease while massaging. I feel like serving others but I am cursed.… I get a fever and swelling which becomes worse during the cold.”*

### 3.2. Healthcare-Seeking Behaviors

#### 3.2.1. Seeking Traditional and Conventional Health Care

Respondents were asked what kind of services they had taken for the treatment of Lymphoedema. Most of the respondents said that they used traditional and home-based care in the first episodes of Lymphoedema. Faith/beliefs such as Dhami/Jhakri, avoidance of food, astrologists and* Pandits*, and home-based treatments such as hot water and sponging had been used. A man who had sought care from traditional healers said,

*“… For treatment, at first I approached Dhami/Jhakri and even performed Naag Puja (a Hindu ritual worshipping snake god). Later I went to Amppipal Hospital (local hospital) where Lymphoedema was diagnosed. Following this, I took medicine but did not get cured.”*

Traditional medical remedies such as applying neem (*Azadirachta indica*) leaves and leeches over the swollen area were reported. A man who had used traditional practices said,

*“… I am a pure vegetarian. I preferred home remedies and, therefore, took garlic and cow urine followed by hot sponging but nothing cured me.”*

Most of the respondents mentioned that they had frequently used the analgesics, DEC, diuretics, NSAIDs, Vitamins tablets, antifungal creams, antibacterial creams, and antibiotics bought from medical shops on prescription from health practitioners. Some had never been counseled by health workers. Only few had received treatment for Lymphoedema from conventional health care. There were many hydrocele patients aspiring to the treatment for hydrocele but actually did not have hydrocelectomy. However, most pricked the hydrocele with sharp objects to drain out the fluid. The high cost of surgery and lack of money and fear of death, as well as impotence and/or sterility, were reported as the reasons why they did not have hydrocelectomy. A man who have sought health care said,

*“… I went to Delhi (India) where Lymphoedema was diagnosed and I took medicine, but still I am not cured. I have already spent too much money. Now, I don't have money for operation (hydrocelectomy).”*

#### 3.2.2. Seeking Mental Health Care

Respondents reported various psychological problems such as anxiety, worry and stress, depression, low self-esteem, feeling weak, and fear of being abandoned. A middle aged woman said,

*“… due to this disease, I got married to a poor family… I feel anxious and depressed due to my condition. My family doesn't support me well. I am afraid that my husband will leave me to marry other women”.*

Females were worried more about transmitting the disease to children compared to their counter parts. A middle aged woman said,

*“… I have never visited a doctor to treat this problem. I am worried that it would transmit to my offspring.”*

More males reported painful sexual intercourse and psychological problems. However, none had taken treatment or counseling for the management of the problem. A man having a Lymphoedema in legs and hydrocele said,

*“… I had a very painful sex last month. After that I have refrained sex. My wife complained me for not having sex.”*

### 3.3. Self-Care Practices

Only a quarter of the study population was aware and a few of them had been practicing standard foot care practices like soap water washing, elevation and exercise of the affected limb, nail hygiene, trauma reduction and care for the entry lesion, antiseptics, and use of footwear on a regular basis. The majority of the respondents had been practicing washing the affected limbs only. Among the six foot care measures asked, only two had done at least one or two measures other than washing. Wearing specially made foot wears was observed in none of the patients. They did not have knowledge of foot care practices; patients said when asked about why they did not practice such practices. Application of medicinal bandages, herbal preparations, and leeches on the swollen area was reported. In rare cases, patients used herbal preparations orally, or smeared them on affected parts, or those preparations were given as an enema, and even scarification of affected parts was reported. Respondents reported that they were able to wear clothes, walk, and bathe, but had a feeding problem, had trouble in doing agricultural work, and had difficulty in using a toilet and difficulty in brushing their teeth. A man with severe filarial Lymphoedema in hand said,

*“… I can't eat food with my hands so I use a spoon… I am able to use a toilet, wear clothes, take a bath and take care of my body.”*

However, many said that they were not assisted by family member in feeding, using toilet, and wearing clothes. A middle-aged woman said,

*“… although I can do my personal hygiene, I have trouble in going to the toilet. Sometimes due to swelling, I can't even do my daily chores but as I have no one in the family to assist me, I have to do them anyway with pain….”*

Some respondents said that they abstained from certain types of foods such as tea, sour items, onion, brinjal, Kuvindo (vegetable), salt, fish, and yoghurt. They believed that these types of foods would deteriorate their physical condition. A woman said,

*“I had not taken Brinjal and Kuvindo for the past two years. I have restricted yoghurt and salt consumption. Earlier, when I had them, my legs were swelled.”*

## 4. Discussion

This study was conducted in a small geographic area with diverse ethnic population in each district. Four different local terminologies were found referring to the names of the disease. The identified terminologies can be used to develop information, education, and communication (IEC) materials that are locally appropriate.

The majority of the respondents did not believe that the parasite in the body due to mosquito bites is the real cause of the disease. Quite the opposite, they attributed the disease to their past work and physical activity. Study conducted in South India reported that only 9% of people having LF and 20% of those without the disease knew that filariasis is caused through mosquito bites; the rest attributed it to many other causes [22]. Similarly around 42% of people during the pre-MDA period had accurate knowledge of the association of LF with mosquito bites [23]. In general, people did not accept the mosquito theory of transmission, but they believed in other physical, spiritual, and hereditary causes [24]. The current study showed that only a few knew about the preventive measures against the disease. About 83% of the affected and 87% of the unaffected individuals either were uncertain or felt that filariasis is not preventable [22]. Krentel et al. reported that the knowledge of the cause of the disease and its prevention is important for compliance with mass drug administration [25]. Hence awareness activities during mass drug administration campaigns need to be emphasized for disseminating messages related to the cause and prevention of Lymphoedema.

Lymphoedema is debilitating in humans; it has several manifestations, including Lymphoedema and hydrocele [26]. The study participants had swelling of the legs and the hands and some even presented with knobs, lesions, and skin folds in the body. A study conducted in Ghana found that the most common illnesses in the study communities were joint pains, Lymphoedema, stomach pains, hernia: hydrocele, measles, malaria: fever, headache, dizziness, eye problems, diarrhoea, ADL attacks, and waist pains [24]. The greater the degree of Lymphoedema, the lesser the quality of life they have. The morbidity management is one of the objectives of the national LF elimination program in Nepal [11]; there are no activities run at community level to provide treatment, care, and support for people with Lymphoedema.

We found that the majority of the respondents visited traditional faith healers prior to their seeking modern medicine. Ayurveda, Homeopathy, Unani, and Amchi are the major alternative medicines that have long been practiced in Nepal [27]. Also, the Ayurvedic treatment was also practised for Lymphoedema management (25%) followed by home remedies and acupuncture in Sri Lanka [28]. Dhami and Jhakri are the faith-based traditional healers [29]. They are the first point of contact of care in rural areas of the country [30]. There are public health programs on reproductive health and child morbidity to train the traditional faith healers to refer patients coming to them to the health centers [31]. This approach can be replicated for Lymphoedema management activities as well. Therefore, trainings should be provided for alternative medicine providers so that they can provide counseling to manage Lymphoedema and refer the cases to health centers.

Self-care for the disease reduces health care utilization and potentially yields monetary benefits to a health plan [32]. In conformity with an earlier study in India, only a few respondents had practiced standard foot care practices [33]. Washing affected limbs was common among the respondents, though it cannot be linked to Lymphoedema care, as washing hands and feet is common in the Hindu rituals [33]. Respondents were not wearing larger size footwear and none of them reported massaging the affected limbs. A regular footwear might not prevent the individuals from external injuries; therefore, a larger size footwear should be recommended. Massaging affected limbs relieves pain and an elevation exercise prevents accumulation of fluids in affected limbs. Both should be recommended [7]. Standard foot care practices can be incorporated into routine counseling services in health centers in endemic communities. Engaging in self-care as part of daily disease management is crucial in villages in Nepal on account of villagers' poor access to health centers. Lymphoedema cases might have difficulty in traveling to health centers for their treatment because in most of the cases, the health centers are more than thirty minutes' walk from their place of residence. Thus, home-based management with a greater emphasis placed on foot care aimed at Lymphoedema patients needs to be promoted. It is found that hydrocoele was the commonest manifestation among the LF cases in this study. In line with a study from Ghana, most of the hydrocoele patients mentioned that the main reasons for refusing hydrocelectomy were the high cost of surgery and lack of money and to some extent fear of death and impotence and/or sterility that might result from the operation [9].

Just as the previous studies reported, food taboos like refraining from taking certain foods and drinks [9], tea, sour items, onion, brinjal,* Kuvindo* (vegetable), salt, fish, and yoghurt were reported. Abstinence reduces the supply of nutrients and minerals in the body, possibly leading to poor health. Health workers then have an additional role in launching food awareness activities.

A previous literature reported Lymphoedema is a significant cause of psychological morbidity [9]. Psychological problems reported by the respondents in this study are in strong agreement with those in previous studies. A review literature reported fear, anxiety, frustration, and distress [3] in people with Lymphoedema. In our study population, illiteracy, lack of treatment, and poor or no counseling might be the causes of the reported cases having fear of transmitting disease to their children. While it was found that the respondents had some psychological conditions, the importance of social participation by those who have this condition needs to be emphasized more by peripheral health workers in Nepal. Previous studies reported that the role of social participation is important in rehabilitation and reduction of psychological trauma and problems [34].

This study is perhaps the first of its kind to report health-seeking behaviors and self-care practices of people living with filarial Lymphoedema in Nepal. We realize that the study has a number of limitations. Subjects with mild and moderate forms of filarial Lymphoedema might not have been studied well in this study, for they did not participate for reasons, such as in part the hidden nature of the disease and in part our inability to indentify them. The health-seeking behavior and self-care practices reported in this study might not represent all of the practices across the diverse topography and people in Nepal.

## 5. Conclusions 

We identified two terminologies each signifying Lymphoedema of hands and feet. Lymphoedema in the limbs and hydrocele were found to be major health problems in the studied communities. The majority of the respondents did not believe that the parasite in the body due to mosquito bites is the real cause of the disease. The traditional health care providers were the first contact of care for the majority of respondents. They need to be trained to manage and refer Lymphoedema patients to health facilities. Only a few patients had been practicing standard foot care practices. Active programmes for surgical management Lymphoedema and standard foot care practices should be emphasized in lymphatic filariasis elimination programme by involving government bodies, NGOs, and other community based organizations.
